# Expert ratings of job demand and job control as predictors of injury and musculoskeletal disorder risk in a manufacturing cohort

**DOI:** 10.1136/oemed-2015-102831

**Published:** 2015-07-10

**Authors:** Linda F Cantley, Baylah Tessier-Sherman, Martin D Slade, Deron Galusha, Mark R Cullen

**Affiliations:** 1 Occupational and Environmental Medicine Program, Yale University School of Medicine, New Haven, Connecticut, USA; 2 Stanford Center for Population Health Sciences, Stanford University School of Medicine, Stanford, California, USA

## Abstract

**Objective:**

To examine associations between workplace injury and musculoskeletal disorder (MSD) risk and expert ratings of job-level psychosocial demand and job control, adjusting for job-level physical demand.

**Methods:**

Among a cohort of 9260 aluminium manufacturing workers in jobs for which expert ratings of job-level physical and psychological demand and control were obtained during the 2 years following rating obtainment, multivariate mixed effects models were used to estimate relative risk (RR) of minor injury and minor MSD, serious injury and MSD, minor MSD only and serious MSD only by tertile of demand and control, adjusting for physical demand as well as other recognised risk factors.

**Results:**

Compared with workers in jobs rated as having low psychological demand, workers in jobs with high psychological demand had 49% greater risk of serious injury and serious MSD requiring medical treatment, work restrictions or lost work time (RR=1.49; 95% CI 1.10 to 2.01). Workers in jobs rated as having low control displayed increased risk for minor injury and minor MSD (RR=1.45; 95% CI 1.12 to 1.87) compared with those in jobs rated as having high control.

**Conclusions:**

Using expert ratings of job-level exposures, this study provides evidence that psychological job demand and job control contribute independently to injury and MSD risk in a blue-collar manufacturing cohort, and emphasises the importance of monitoring psychosocial workplace exposures in addition to physical workplace exposures to promote worker health and safety.

**What this paper adds**

- Psychosocial hazards in the workplace are recognised as emerging threats to worker well-being, but the bulk of research examining adverse health and safety outcomes associated with these hazards has relied on worker self-reports of exposures, outcomes or both, raising concerns that individual psychological traits may confound reported results.
- Applying an expert-based job exposure matrix for assessment of physical job demand, psychological job demand and job control, multivariate mixed effects models were used to examine associations between the expert assessments of job-level demand and control, and acute injury, and musculoskeletal disorder (MSD) risk, adjusting for manufacturing process, plant and individual-level covariates.
- Workers in jobs within the highest tertile of rated job demand showed elevated risk of serious injury and serious MSD, while those in the lowest tertile of rated job control had increased risk for minor injury and minor MSD. No interactions were observed between physical demand, psychological demand and job control, suggesting independent contribution of these exposures to injury and MSD risk.

With technological changes, new management ideologies, and increases in global competition for manufacturing, considerable changes in the organisation and management of work have occurred, requiring employees to work harder, faster, more productively and with less influence over daily work tasks.^1^ Psychosocial hazards arising from these changing work demands have been recognised as emerging threats to physical and mental health,^2^ with mounting evidence suggesting associations between these stressors and work-related injury,^3^ ^4^ as well as musculoskeletal disorder (MSD) risk.^5–7^

Work-related injuries and MSDs remain a public health concern worldwide with potentially severe consequences for both workers and employers.^8^ The contribution of physical hazards arising from job tasks, workplace environment, tools and equipment,^9–11^ and of poor psychosocial working conditions to work-related injury and MSD risk has been demonstrated in a number of different worker cohorts.^3^ ^5–7^ While the contributory role of psychosocial work demands in cumulative MSD risk is better recognised,^5^ ^6^ ^12^ high psychological demands, alone or in combination with low decision latitude, could alter worker vigilance to safety precautions and worker risk recognition, as well as increase worker error rates, each of which may elevate risk for acute musculoskeletal as well as non-musculoskeletal injury. Despite recognition that physical and psychosocial workplace hazards often coexist and may be correlated,^2^ ^12^ ^13^ in addition to evidence that each contributes to injury and MSD risk when considered separately, epidemiological studies examining concurrent physical and psychosocial workplace stressors in predicting risk have been limited.^9^ ^14^ ^15^ This gap represents a particular shortcoming given reports suggesting a synergistic interaction between physical and psychosocial demands that may further increase risk for injury or MSDs,^9^ ^16^ ^17^ and reports proposing that psychosocial demands may contribute to stress which could alter physiological and behavioural reactions.^16^ ^18^ Moreover, associations between these different sources of job strain and occupational injury and MSD risk among hourly manufacturing workers has been explored in limited reports,^4^ ^19^ ^20^ but has yet to be fully examined, and is another gap to be filled.

Most research examining associations between psychosocial job demands and injury or MSD risk has relied on self-report by job incumbents.^3^ ^21^ Use of self-reported measures to examine such associations has raised concern that psychological traits and states of the individual may be the ultimate determinant for observed associations rather than objective aspects of the job.^22^ ^23^ Moreover, a focus on individual-level perceptions of exposure may foster individual-level rather than broader work cultural or organisational interventions that could potentially benefit more workers. To mitigate these concerns, some investigators have used expert ratings of jobs by non-incumbents, either alone or in combination with subjective individually rated measures.^24–26^ Because subjective ratings by job incumbents are time-consuming and can be cumbersome to obtain, their practicality and utility in modern workplaces with greater time constraints and increasingly scarce resources is limited. Expert job-level ratings of workplace demands may offer a more cost effective and efficient mechanism to examine these exposures as determinants of workplace injury risk and better illuminate opportunities for exposure reduction to improve safety outcomes.

As companies in the manufacturing sector strive to maintain economic viability in a globally competitive marketplace, more thorough examination of associations between concomitant physical and psychosocial workplace exposures and injury and MSD risk is a critical step toward improving worker health and safety. The primary objective of this study is to assess expert ratings of physical demand, psychological demand, and job control as predictors of injury and MSD risk among a prospective cohort of aluminium manufacturing workers. We hypothesise that expert ratings of exposure assigned by job title using a job exposure matrix will predict risk for work-related injury and MSDs.

## Methods

### Study cohort and data

Data used for this study is available through a longstanding collaboration between the investigators and a multinational aluminium manufacturing company. All production and maintenance workers at eight aluminium manufacturing plants, covering a range of processes including smelting, fabricating and forging, who worked between 1 January 2004 and 31 December 2005 in jobs for which expert ratings of physical demand, heat exposure, psychological demand and job control were obtained in late 2003 comprised the study cohort.

We constructed job histories for each worker during the 2-year study period immediately following rating obtainment, calculated active time worked in each job held, and obtained demographic characteristics for the cohort using the human resources database, which has been well described in previous reports.^27^ ^28^ The study company's real-time incident surveillance database, which was established in 1989 and previously described,^27^ requires reporting of all incidents including minor incidents that necessitate first aid only. Using the ‘nature of injury’ variable, we identified all incidents resulting in injury or MSD, and constructed incident histories for each worker in each job held during the 2-year study period. The ‘case-type’ variable was used to differentiate minor incidents requiring only first aid from the more serious incidents that resulted in medical treatment, restricted work or lost work time. All minor and serious injuries, and minor and serious MSD incidents for each person-job occurring between 1 January 2004 and 31 December 2005 were included for analysis. Types of injury included for analysis comprised lacerations, contusions, burns, amputations, etc, while MSDs included instantaneous strains and sprains, non-instantaneous strain and sprains, non-instantaneous low back disorder, etc.

Separate analyses were conducted to examine each of the following outcomes: minor injury and minor MSD requiring first aid only; serious injury and serious MSD that required medical treatment, restricted work or lost work time; the subset of minor MSDs requiring first aid only, and the subset of serious MSDs that required medical treatment, restricted work or lost work time.

Expert ratings of job-level physical demand, heat exposure, psychological demand and job control were obtained in late 2003 from one senior health and safety professional at each of eight plant locations using a pilot job demand survey (table 1). The survey was introduced in an attempt to better understand the various aspects of company jobs, not specifically to address injury risk reduction activities. Each rater was familiar with all plant departments and jobs, received standardised instruction via teleconference as well as written instructions prior to survey completion from the lead author, and received a complete listing of plant job titles by department for rating. The expert raters assigned exposure ratings to the plant jobs based on their job content knowledge rather than individual worker assessments. Across the eight plant locations, the number of manufacturing and maintenance jobs ranged from 32 to 57 job titles per plant. Notably, none of the raters represented incumbents or supervisors of rated plant jobs and had never been responsible for reporting into or maintaining the company's incident surveillance database.

**Table 1:** Job demand survey

1. *Physical Demand*: Select the degree of physical activity that best describes the job based on the following definitions			
Physical Demand Level	How much force must be exerted to lift, carry, push, pull or otherwise move objects, and how frequently is that exertion of force required by the job?		
	Occasional (0–33% of the workday)	Frequent (34–66% of the workday)	Constant (67–100% of the workday)
Sedentary	10 lbs	Negligible	Negligible
	Jobs are sedentary if walking and standing are required only occasionally and all other sedentary criteria are met		
Light	20 lbs	10 lbs and/or walk and/or stand with operation of controls	Negligible and/or operate controls while seated
	If negligible force exertion is required, a job should be rated Light if it requires: (1) walking or standing to a significant degree; (2) sitting most of the time but entails pushing and/or pulling of arm or leg controls and/or (3) working at a production rate pace entailing the constant pushing and/or pulling of materials		
Medium	20–50 lbs	10–25 lbs	10 lbs
	Physical Demand requirements are in excess of those for Light work		
Heavy	50–100 lbs	25–50 lbs	10–20 lbs
	Physical Demand requirements are in excess of those for Medium work		
Very Heavy	> 100 lbs	>50 lbs	>20 lbs
	Physical Demand requirements are in excess of those for Heavy work		
Response Categories for Analytic Models: Sedentary/Light (Referent) Medium Heavy/Very Heavy			
2. *Heat Exposure:* Estimate the duration of significant or unacceptable exposure to heat for employees in the job during the season(s) when heat is a concern: Not at all, Occasionally, About half the time, Almost all the time			
Response Categories for Analytic Models: Never (Referent) Up to half the time More than half the time			
*Psychological Demand*: 12-point scale from 1 (often) to 12 (never)			
3. How often does the job involve working very fast? 4. How often is it extremely important to do the work without mistakes? 5. How often does the job demand simultaneous or consecutive completion of tasks that are difficult to combine?			
Response Categories for Analytic Models: Summed composite score yielding tertiles of: Low Demand (Referent) Moderate Demand High Demand			
*Decision latitude/Job control*: 12-point scale from 1 (often) to 12 (never)			
6. How often does the job permit complete discretion and independence in determining how the work is to be done? 7. How often does the job permit complete discretion and independence in determining when the work is to be done?			
Response Categories for Analytic Models: Summed composite score yielding tertiles of: Low Control Moderate Control High Control (Referent)			

Overall physical job demand was rated using the US Department of Labor physical demand characteristics of work classification scheme with the raters classifying each job into the well-defined categories of Sedentary, Light, Medium, Heavy or Very Heavy.^29^ Heat exposure was rated using a four-point frequency scale ranging from 1 (not at all) to 4 (almost all the time). Psychological job demand and decision latitude (job control) were rated on a 12-point scale ranging from 1 (often) to 12 (never) using questions similar to those used in the Whitehall II study,^25^ ^30^ based on the demand control/job strain model.^31^ ^32^ Raters were instructed to respond according to what each specific job required.

Because very few of the plant-jobs received physical demand ratings of sedentary or very heavy, the physical demand ratings were collapsed into three categories: sedentary/light, medium or heavy/very heavy for analysis. The heat exposure ratings were collapsed into three categories: more than half the time, up to half the time or never. Ratings for the three psychological demand questions and the two job control questions were summed to yield a composite demand and control score for each job. Tertiles were used to designate job demand and job control as high, moderate, or low as was done in the Whitehall II study.^24^ ^30^ Ratings of each exposure variable were assigned by job title to each member of the study cohort for each job held during the 2-year study period.

### Statistical analysis

Descriptive statistics for the study cohort were calculated. Unadjusted minor incident (injury and MSD), serious incident (injury and MSD), minor MSD only, and serious MSD only rates were calculated for each category of physical demand and heat exposure, and each tertile of psychological demand and job control by dividing the number of category-specific events by the summed number of category-specific person-years and multiplying by 100. Spearman rank correlation coefficients were calculated to assess collinearity between exposure variables.

Multivariate generalised linear mixed models were used to estimate relative risks (RR) for injury along with corresponding 95% CIs for physical demand category (sedentary/light, medium, or heavy/very heavy) with sedentary/light used as the referent, heat exposure category (never, <half the time or >half the time) with never used as the referent, psychological demand tertile (high, moderate or low) with low demand used as the referent and job control tertile (high, moderate or low) with high control used as the referent. Multivariate models were additionally adjusted for age, sex, job tenure <1 year, race/ethnicity, manufacturing process and plant. For the minor incident outcomes: minor injury and minor MSD, and minor MSD only, a Poisson distribution with a log link and offset of the log person-days which contributed to each job was chosen. To better fit the distribution of the serious injury and MSD and serious MSD only outcomes among the cohort, we selected a binomial distribution with a logit link for those outcomes. Summed person time contributed to each job was the injury rate denominator for the serious incident and serious MSD outcomes. Random intercepts for person clustered within job, and job clustered within plant were incorporated to allow for within and between person and job variation, and account for correlation resulting from clustering. An unstructured covariance structure was specified.^33^ All p values were two sided, and a value of less than α=0.05 was considered statistically significant. All interactions between physical demand, heat exposure, psychological demand, and job control were examined. Finally, excess fractions of outcomes were calculated for each statistically significant exposure.

Statistical analyses were performed with SAS V.9.3 (SAS Institute, Cary, North Carolina, USA). This study received ethical approval from the Yale University School of Medicine Human Investigations Committee.

## Results

There were 391 unique plant-job combinations for the eight study plants for which expert ratings of physical and psychological job demand, heat exposure and job control were obtained. During the 2-year period immediately following rating obtainment, 9260 production and maintenance workers contributed 15 232 person-years to rated jobs within the study plants. Eighty per cent of the study cohort held only one job during the 2-year study period, while 15% held two jobs and 5% held more than two jobs. Table 2 displays descriptive statistics for the cohort at the start of the study period and the distribution of all exposures of interest. Spearman rank correlation coefficients indicated low correlation between rated exposure variables with the highest correlation observed between ratings of physical demand and heat exposure (r=0.31), and the lowest correlations observed between psychological demand and physical demand (r=−0.02), and between job control and heat exposure (r=−0.02). Ratings of psychological demand and job control had a correlation of r=−0.19. Among the study cohort—90% male and 86% white—1641 persons who worked in 1683 person-jobs sustained 2060 minor injuries and minor MSDs requiring first aid treatment only, while 615 workers who worked in 620 jobs sustained 658 serious injuries and serious MSDs that required medical treatment, work restrictions or lost work time. Considering the subset of MSDs only, 674 persons who worked in 685 person-jobs sustained 747 minor MSDs, requiring first aid only, while 256 persons who worked in 259 person-jobs sustained 264 more serious MSDs requiring medical treatment, work restrictions, or lost work time during the study period.

**Table 2:** Descriptive statistics at baseline and distribution of individual-level covariates and job-level exposures by tertile of physical demand, psychological demand and job control for 9260 persons who worked in 11 722 person-jobs 2004–2005

	Full cohort at baseline	Physical demand			Psychological demand			Job control		
	9260 persons	11 722 Person-jobs			11 722 Person-jobs			11 722 Person-jobs		
		Heavy	Medium	Light	High	Moderate	Low	High	Moderate	Low
	N (%)	N (%)	N (%)	N (%)	N (%)	N (%)	N (%)	N (%)	N (%)	N (%)
Person-jobs	11 722 (100)	3953 (100)	5549 (100)	2220 (100)	3801 (100)	3916 (100)	4005 (100)	3460 (100)	3667 (100)	4595 (100)
Age (years), mean (SD)	45.9 (10.3)	43.6 (11.2)	45.8 (10.0)	45.5 (9.4)	43.7 (10.7)	46.4 (10.3)	44.8 (9.9)	48.6 (9.3)	45.2 (10.4)	42.0 (10.2)
Tenure (years), mean (SD)	17.0 (12.3)	14.7 (13.0)	16.3 (12.0)	16.5 (11.4)	14.4 (11.8)	18.0 (12.8)	15.1 (11.8)	19.7 (12.3)	16.4 (12.5)	12.5 (11.1)
Male sex	8314 (90)	3681 (93)	4828 (87)	1952 (88)	3434 (90)	3569 (91)	3458 (86)	3102 (90)	3316 (90)	4043 (88)
Race/ethnicity (white)	7999 (86)	3426 (87)	4838 (87)	1808 (81)	3291 (87)	3494 (89)	3287 (82)	3007 (87)	3129 (85)	3936 (86)
Job tenure <1 year	2486 (27)	1441 (36)	1998 (36)	901 (41)	1468 (39)	1366 (35)	1506 (38)	927 (27)	1424 (39)	1989 (43)
Physical demand										
Heavy/very heavy	3320 (36)	.	.	.	920 (24)	1993 (51)	1040 (26)	952 (27)	1348 (37)	1653 (36)
Medium	4286 (46)	.	.	.	2164 (57)	1258 (32)	2127 (53)	1674 (48)	1671 (45)	2204 (48)
Sedentary/light	1654 (18)	.	.	.	717 (19)	665 (17)	838 (21)	834 (24)	648 (18)	738 (16)
Psychological demand										
High	2989 (32)	920 (23)	2164 (39)	717 (32)	.	.	.	1492 (43)	1111 (30)	1198 (26)
Moderate	3226 (35)	1993 (51)	1258 (23)	665 (30)	.	.	.	1352 (39)	1219 (33)	1345 (29)
Low	3045 (33)	1040 (26)	2127 (38)	838 (38)	.	.	.	616 (18)	1337 (36)	2052 (45)
Job control										
High	2952 (32)	952 (24)	1674 (30)	834 (38)	1492 (39)	1352 (35)	616 (15)	.	.	.
Moderate	2896 (31)	1348 (34)	1671 (30)	648 (29)	1111 (29)	1219 (31)	1337 (33)	.	.	.
Low	3412 (37)	1653 (42)	2204 (40)	738 (33)	1198 (31)	1345 (34)	2052 (51)	.	.	.
Heat										
Almost always	1632 (18)	1369 (35)	552 (10)	185 (8)	1244 (33)	560 (14)	302 (7)	307 (9)	883 (24)	916 (20)
Up to half the time	4518 (49)	1734 (44)	3084 (56)	890 (40)	1706 (45)	2168 (55)	1835 (46)	2277 (66)	2108 (57)	1323 (29)
Never	3110 (33)	850 (21)	1913 (34)	1145 (52)	852 (22)	1188 (30)	1868 (47)	878 (25)	676 (18)	2356 (51)

Table 3 displays the unadjusted injury and MSD rates by category of physical demand and heat exposure and tertile of psychological demand and job control. The unadjusted rate for each outcome examined (minor injuries and minor MSDs, serious injuries and serious MSDs, minor MSDs only or serious MSDs only) was highest for jobs rated as heavy/very heavy physical demand (15.52, 5.57, 6.07 and 2.57, respectively) compared to those rated as light/sedentary or medium physical demand and for jobs rated as exposed to heat more than half the time (22.67, 6.49, 9.09 and 2.34, respectively) compared to jobs rated as less frequently or never exposed to heat. Similarly, the unadjusted rate for each outcome was higher for jobs with high psychological demand (15.08, 5.00, 5.50 and 1.88, respectively) compared to those with moderate or low psychological demand. Jobs having low ratings for job control had the highest serious injury and serious MSD rate, and serious MSD only rate (4.82 and 1.94, respectively) while jobs rated as having moderate job control had the highest minor injury and minor MSD rate, and minor MSD only rate (15.33 and 5.82, respectively).

**Table 3:** Unadjusted injury rates by physical demand and heat exposure category, psychological demand and job control tertile per 100 person-years

	First aid injury and MSD n=2060	Serious injury, and MSD n=658	First aid MSD only n=747	Serious MSD only n=264
	n (rate)	n (rate)	n (rate)	n (rate)
Sedentary/light physical demand	287 (10.28)	92 (3.30)	99 (3.55)	36 (1.29)
Medium physical demand	963 (13.34)	275 (3.81)	331 (4.58)	94 (1.30)
Heavy/very heavy physical	810 (15.52)	291 (5.57)	317 (6.07)	134 (2.57)
Never exposed to heat	492 (9.56)	191 (3.71)	142 (2.76)	69 (1.34)
Exposed to heat <half the time	1044 (13.43)	317 (4.08)	395 (5.08)	141 (1.81)
Exposed to heat >half the time	524 (22.67)	150 (6.49)	210 (9.09)	54 (2.34)
Low psychological demand	609 (11.92)	192 (3.76)	222 (5.01)	77 (1.51)
Moderate psychological demand	721 (13.64)	224 (4.24)	256 (4.82)	96 (1.82)
High psychological demand	730 (15.08)	242 (5.00)	269 (5.50)	91 (1.88)
High job control	614 (11.82)	202 (3.89)	233 (4.49)	74 (1.43)
Moderate job control	709 (15.33)	195 (4.22)	269 (5.82)	85 (1.84)
Low job control	737 (13.61)	261 (4.82)	245 (4.52)	105 (1.94)

MSD, musculoskeletal disorder.

The results of the multivariate random intercept models simultaneously adjusted for physical demand, heat exposure, psychological demand, job control, age, sex, race/ethnicity, job tenure <1 year, plant and manufacturing process are shown in table 4. Physical job demand was associated with increased risk for each outcome examined in a monotonic exposure response pattern, although only heavy/very heavy physical demand attained statistical significance for the minor MSD only and serious MSD only outcomes. Compared to workers in jobs for which physical demand was rated as sedentary/light, those in jobs rated as heavy/very heavy physical demand had elevated risk for all outcomes with a near-doubling of risk for the subset of serious MSDs (RR 1.92; 95% CI 1.11 to 3.31). Higher frequency of heat exposure was associated with increased risk for minor injury and minor MSDs, as well as the subset of minor MSDs only in a monotonic exposure response pattern with jobs rated as exposed to heat more than half the time conferring nearly twice the risk for minor injuries and MSDs (RR 1.78, 95% CI 1.27 to 2.49), and minor MSDs only (RR 1.87, 95% CI 1.19 to 2.92) compared to jobs never exposed to heat. RRs were elevated for the serious injury and serious MSD outcome as well as the subset of serious MSDs only among those in jobs exposed to heat more than half the time, but did not attain statistical significance. High psychological demand was associated with increased risk for serious injury and serious MSD (RR 1.49, 95% CI 1.10 to 2.01) and the subset of serious MSDs only, although the latter did not achieve statistical significance. Psychological demand did not elevate risk for either of the minor incident outcomes. The fraction of serious injury and serious MSD outcomes associated with high psychological demand that could be mitigated through reductions in psychological demand was 11.5%.

**Table 4:** Fully adjusted multivariate mixed model results with random intercepts for job within plant and person within job

	First aid injury and first aid MSD (n=2060)			Serious injury and serious MSD (n=658)			First aid MSD only (n=747)			Serious MSD only (n=264)		
	RR*	95% CI	Pr>F	RR*	95% CI	Pr>F	RR*	95% CI	Pr>F	RR*	95% CI	Pr>F
Sedentary/light physical demand	1.00	Reference	0.0297	1.00	Reference	0.0052	1.00	Reference	0.0493	1.00	Reference	0.0206
Medium physical demand	1.26	1.02 to 1.55		1.41	1.04 to 1.90		1.26	0.95 to 1.69		1.13	0.71 to 1.79	
Heavy/very heavy physical demand	1.44	1.09 to 1.91		1.88	1.28 to 2.75		1.58	1.10 to 2.29		1.92	1.11 to 3.31	
Never exposed to heat	1.00	Reference	0.0034	1.00	Reference	0.1250	1.00	Reference	0.0165	1.00	Reference	0.2936
Exposed to heat up to half the time	1.38	1.07 to 1.77		1.21	0.89 to 1.66		1.57	1.11 to 2.22		1.43	0.91 to 2.23	
Almost always exposed to heat	1.78	1.27 to 2.49		1.57	1.02 to 2.42		1.87	1.19 to 2.92		1.38	0.73 to 2.61	
Low psychological demand	1.00	Reference	0.5428	1.00	Reference	0.0374	1.00	Reference	0.0929	1.00	Reference	0.0672
Moderate psychological demand	0.92	0.74 to 1.14		1.21	0.90 to 1.62		0.75	0.57 to 1.00		1.05	0.69 to 1.59	
High psychological demand	1.04	0.82 to 1.31		1.49	1.10 to 2.01		0.95	0.71 to 1.29		1.60	1.04 to 2.47	
High job control	1.00	Reference	0.0071	1.00	Reference	0.0937	1.00	Reference	0.2408	1.00	Reference	0.0977
Moderate job control	1.37	1.10 to 1.79		1.21	0.90 to 1.62		1.22	0.92 to 1.60		1.49	0.97 to 2.27	
Low job control	1.45	1.12 to 1.87		1.44	1.04 to 2.01		1.32	0.94 to 1.85		1.63	1.02 to 2.61	
Race/ethnicity white	1.00	Reference	0.5441	1.00	Reference	0.8664	1.00	Reference	0.1843	1.00	Reference	0.6487
Other	1.08	0.70 to 1.66		0.91	0.40 to 2.06		1.55	0.85 to 2.83		0.39	0.06 to 2.48	
Hispanic	1.18	0.94 to 1.48		1.12	0.77 to 1.63		1.34	0.96 to 1.88		1.23	0.70 to 2.18	
Black	0.99	0.84 to 1.17		1.08	0.84 to 1.38		1.05	0.82 to 1.36		1.07	0.74 to 1.56	
Job tenure <1 year	1.47	1.30 to 1.65	<0.0001	1.33	1.08 to 1.64	0.0070	1.41	1.17 to 1.72	0.0004	1.55	1.13 to 2.12	0.0067
Age	0.99	0.98 to 0.99	0.0001	0.99	0.99 to 1.00	0.2615	0.99	0.98 to 1.00	0.0111	0.99	0.98 to 1.00	0.1426
Female sex	1.51	1.31 to 1.73	<0.0001	1.55	1.23 to 1.93	0.0001	1.26	1.00 to 1.59	0.0462	1.75	1.25 to 2.46	0.0011

Psychological demand=(working fast+working without mistakes+working with conflicting demands).

Job control=(control over how job is done+control over when job is done).

*Adjusted for physical demand, heat exposure, psychological demand, job control, sex, age, job tenure <1 year, plant, manufacturing process, race/ethnicity with random intercepts for job within plant and person-within-job.

MSD, musculoskeletal disorder; RR, relative risk; Pr>F, significance test for fixed effects.

Compared to workers in jobs rated as having a high level of job control, low job control was associated with increased risk for minor injury and minor MSD (RR 1.45, 95% CI 1.12 to 1.87). RR estimates for the lowest tertile of job control were also elevated for the serious injury and serious MSD outcome as well as the serious MSDs only outcome though the test for trend indicated no statistically significant difference between exposure categories. Moderate job control was associated with increased risk only for the minor injury and minor MSD outcome (RR 1.37, 95% CI 1.10 to 1.79). The estimated fraction of minor injury and minor MSDs that could be reduced through increases in job-level decision latitude was 9%.

Both female sex and job tenure less than 1 year were consistently associated with increased risk for each of the outcomes examined, while race/ethnicity did not predict risk for any of the outcomes. Increasing age was associated with reduced risk for both minor outcomes examined but did not attain statistical significance in predicting more serious outcomes among this cohort. No significant interactions between physical demand, psychological demand, job control or heat exposure were observed.

## Discussion

Our study results are consistent with existing evidence that physical demand, psychological demand and job control are each independent predictors of injury and MSD risk among blue-collar manufacturing workers. Using expert ratings of job-level demand and control, and applying generalised linear mixed models with random intercepts for job within plant and person within job along with adjustment for manufacturing process and individual-level covariates, we show that an increasing level of physical demand is consistently associated with increasing injury and MSD risk. Associations between decreasing levels of job control and increasing levels of psychological demand and injury and MSD risk are less clear. High levels of psychological demand predicted risk for the serious injury and serious MSD outcome, as well as the subset of serious MSDs only, although the trend for the latter did not attain statistical significance at the p<0.05 level. However, psychological demand did not predict risk for the minor outcomes in this cohort. Low levels of job control clearly predicted risk only for the minor injury and minor MSD outcome, although the RR estimates for the serious injury and serious MSD outcome and the subset of serious MSDs only were suggestive of elevated risk for the lowest tertile of job control. We found no significant interactions between expertly rated physical demand, psychological demand and job control, suggesting no synergistic or antagonistic effects of these predictors on outcomes within our study cohort. This provides evidence that the adverse effects of psychosocial workplace hazards are not limited to the subset of workers in the highest strain jobs, that is, jobs conferring a combination of high psychological demand and low job control.

Our findings are consistent with the general theory that psychosocial workplace exposures extend their influence beyond the domain of health, and build on previous, albeit limited evidence that psychosocial demands are important determinants of injury and MSD risk among hourly manufacturing workers,^4^ ^9^ even after controlling for the recognised contribution of physical demand and individual-level covariates.

In partial support of findings from a recent study in which associations between psychosocial job strain and new onset low back pain were observed only among automobile workers with high physical work exposures,^9^ we found that job control and psychological demand predicted injury and MSD risk independent of physical demand. Additionally, our results support observations by Kim *et al*^4^ that high cognitive demands and low job control predict injury risk in manufacturing workers. By contrast with these previous reports, which were limited by reliance on self-reported data for injury occurrence, availability of company maintained incidence data that requires complete reporting regardless of severity allowed us to examine these associations without introducing reporting bias. Thus, our findings provide additional evidence for the strength of association between psychosocial workplace exposures and injury and MSD risk. Interestingly, for none of the examined outcomes did expert ratings of both psychological demand and job control predict risk at a statistically significant level, a finding that diverges from the job strain hypothesis. However, previous studies among hospital personnel^21^ and construction workers^19^ have reported similar findings.

Consistent with findings from previous reports, increasing physical demand predicted increased injury and MSD risk among our study cohort.^27^ ^34^ In addition, female sex and job tenure less than 1 year were each predictive of increased injury and MSD risk, observations previously shown in studies of similar worker cohorts.^35^ ^36^ Notably, the observed association between frequency of heat exposure and risk for injury and MSD in our cohort, an observation that remained despite adjustment for manufacturing process, suggests that ambient heat exposure—beyond that generated by smelting—may be an important predictor of risk and merits further investigation.

Although our approach of using highly experienced, plant-based health and safety professionals to provide ratings of job-level demand and control arguably lends more objectivity than reliance on worker self-report alone, this methodology introduces rater subjectivity, another source of potential bias.^22^ Separate examination of expert ratings and worker self-report of psychosocial job demand has reported weaker associations with cardiovascular outcomes for expert ratings compared to self-reported ratings.^25^ ^37^ This difference in observed associations suggests that expert assessments of the work environment may not fully capture elements meaningful to all individuals.^22^

Interestingly, high correlation has been shown between expert ratings of job control and individual self-report, especially among blue-collar workers, though workers rated their level of job control systematically higher than did the expert raters.^38^ Conversely, substantially lower correlations between expertly rated and self-reported psychological demand have been reported, suggesting perhaps that perceived psychological work demands may vary a great deal between individual workers.^38^ Further study examining concordance/discordance between expert and individual-level ratings of psychological demand and control is warranted to determine future utility as predictors of injury and MSD risk. Notably, none of the raters acted as the plant health and safety manager responsible for plant-wide injury reporting prior to or at the time of the survey; therefore, we do not believe their ratings were likely skewed by prior knowledge of job-level injury history.

This study has several strengths. The large cohort and array of available data allowed for precise job history construction, accurate assignment of job-level exposure ratings and thorough identification of work-related injury and MSD records by job for each member of the study cohort. The completeness of the study company's incident surveillance data, which has been previously validated,^27^ represents a particular strength given widespread concerns about biases due to injury under-reporting. The distribution of minor, first aid only injuries and MSDs by level of job demand and job control was very similar to the distribution for more serious outcomes, minimising concern that our findings result from reporting bias.

Further, available data on several covariates previously shown to predict injury risk allowed us to control for these predictors thereby increasing accuracy of the RR estimates attributed to physical demand, psychological demand and job control. Inclusion of random intercepts for job within plant and person within job in statistical models allowed us to account for correlation resulting from clustering, and provide increased confidence in our study results. Additionally, our use of expert ratings of job-level physical and psychosocial demand offers increased efficiency and cost effectiveness compared to conducting a field survey of individual workers, a potentially appealing feature to companies facing increasing time and resource constraints. This approach could be easily replicated in other workplaces with potential to serve as a catalyst to enhance existing strategies for injury risk mitigation.

Despite these strengths, there are some limitations to this study worthy of mention. We relied on a single expert rater at each of the eight plants, introducing a degree of rater bias inseparable from more general plant differences. While having more than one rater to provide the expert ratings of job-level exposures for each study plant would have been preferable, this approach was not practical because availability of expert raters with sufficient intimate knowledge of job-specific exposures was limited. Although we attempted to control for indistinguishable plant and rater differences by incorporating a fixed effect for plant in our statistical models, a measure of inter-rater reliability would be vastly superior. In addition, because the job-level ratings were obtained at a single time, we were unable to account for any changes in exposures during the study period. However, restricting our study period to the 2 years immediately following rating obtainment likely reduced any misclassification resulting from changes in job-level exposures. Further, previous reports suggest that female workers may perceive psychosocial job demands, particularly decision latitude, differently than their male counterparts performing the same job.^38^ Lacking individual ratings, we are unable to ascertain whether differing perceptions by sex explain the elevation in injury risk consistently observed among female workers in our cohort and previous cohorts from the study company.^36^ Finally, we were unable to control for any differential effect of hours worked, smoking status, or body mass index, each of which has been associated with increased injury risk in previous reports.^3^ ^39^ ^40^

## Conclusion

Using an efficient and cost-effective approach to obtain expert ratings of job demand and control, and adjusting for the contribution of physical workplace exposures as well as plant, manufacturing process and individual-level covariates, our findings highlight the independent contributions of psychological demand and job control to injury and MSD risk in a blue-collar manufacturing cohort. Our results indicate a need for further study to more clearly elucidate associations between psychosocial workplace exposures and injury and MSD risk, as well as potential mechanisms through which these exposures may increase risk. However, regardless of the precise mechanism through which psychosocial workplace exposures increase risk for injury and MSD, these findings, in combination with evidence from previous reports, highlight the importance of monitoring and reducing not only physical but also psychosocial workplace stressors to promote worker health and safety.

## References

[1] Green F Demanding work; the paradox of job quality in the affluent economy. Princeton, NJ: Princeton University Press, 2005.

[2] European Agency for Safety and Health at Work. Expert forecast on emerging psychosocial risks related to occupational health and safety. Luxembourg: Office for Official Publications of the European Communities, 2007.

[3] Swaen GM, van Amelsvoort LP, Bultmann U, et al Psychosocial work characteristics as risk factors for being injured in an occupational accident. J Occup Environ Med 2004;46:521–7. 10.1097/01.jom.0000128150.94272.12 15213513

[4] Kim H-C, Min J-Y, Min K-B, et al Job strain and the risk for occupational injury in small- to medium-sized manufacturing enterprises: a prospective study of 1,209 Korean employees. Am J Ind Med 2009;52:322–30. 10.1002/ajim.20673 19142960

[5] Bongers PM, Ijmker S, van den Heuvel S, et al Epidemiology of work related neck and upper limb problems: psychosocial and personal risk factors (Part I) and effective interventions from a bio behavioural perspective (Part II). J Occup Rehabil 2006;16:272–95. 10.1007/s10926-006-9044-1 16850279

[6] da Costa BR, Vieira ER Risk factors for work-related musculoskeletal disorders: a systematic review of recent longitudinal studies. Am J Ind Med 2010;53:285–323. 10.1002/ajim.20750 19753591

[7] Devereux JJ, Vlachonikolis IG, Buckle PW Epidemiological study to investigate potential interaction between physical and psychosocial factors at work that may increase the risk of symptoms of musculoskeletal disorder of the neck and upper limb. Occup Environ Med 2002;59:269–77. 10.1136/oem.59.4.269 11934955 PMC1740269

[8] Nelson DI, Concha-Barrientos M, Driscoll T, et al The global burden of selected occupational diseases and injury risks: Methodology and summary. Am J Ind Med 2005;48:400–18. 10.1002/ajim.20211 16299700

[9] Vandergrift JL, Gold JE, Hanlon A, et al Physical and psychosocial ergonomic risk factors for low back pain in automobile manufacturing workers. Occup Environ Med 2012;69:29–34. 10.1136/oem.2010.061770 21586759

[10] Mayer J, Kraus T, Ochsmann E Longitudinal evidence for the association between work-related physical exposures and neck and/or shoulder complaints: a systematic review. Int Arch Occup Environ Health 2012;85:587–603. 10.1007/s00420-011-0701-0 22038085

[11] Tawatsupa B, Yiengprugsawan V, Kjellstrom T, et al Association between heat stress and occupational injury among Thai workers: findings of the Thai Cohort Study. Ind Health 2013;51:34–46. 10.2486/indhealth.2012-0138 23411755

[12] Kausto J, Miranda H, Pehkonen I, et al The distribution and co-occurrence of physical and psychosocial risk factors for musculoskeletal disorders in a general working population. Int Arch Occup Environ Health 2011;84:773–88. 10.1007/s00420-010-0597-0 21120664

[13] MacDonald LA, Karasek RA, Punnett L, et al Covariation between workplace physical and psychosocial stressors: evidence and implications for occupational health research and prevention. Ergonomics 2001;44:696–718. 10.1080/00140130119943 11437204

[14] Marras WS, Cutlip RG, Burt SE, et al National occupational research agenda (NORA) future directions in occupational musculoskeletal disorder health research. Appl Ergon 2009;40:15–22. 10.1016/j.apergo.2008.01.018 18462703

[15] Gerr F, Fethke NB, Anton D, et al A prospective study of musculoskeletal outcomes among manufacturing workers: II. Effects of psychosocial stress and work organization factors. Hum Factors 2014;56:178–90. 10.1177/0018720813487201 24669552

[16] Huang GD, Feuerstein M, Sauter SL Occupational stress and work-related upper extremity disorders: concepts and models. Am J Ind Med 2002;41:298–314. 10.1002/ajim.10045 12071486

[17] Waters TR, Dick RB, Krieg EF Trends in work-related musculoskeletal disorders: a comparison of risk factors for symptoms using quality of work life data from the 2002 and 2006 general social survey. J Occup Environ Med 2011;53:1013–24. 10.1097/JOM.0b013e3181fc8493 21278598

[18] Bongers PM de Winter CR, Kompier MA, et al Psychosocial factors at work and musculoskeletal disease. Scand J Work Environ Health 1993;19:297–312. 10.5271/sjweh.1470 8296178

[19] Abbe OO, Harvey CM, Ikuma LH, et al Modeling the relationship between occupational stressors, psychosocial/physical symptoms and injuries in the construction industry. Int J Ind Ergon 2011;41:106–17. 10.1016/j.ergon.2010.12.002

[20] Harris-Adamson C, Eisen EA, Dale AM, et al Personal and workplace psychosocial risk factors for carpal tunnel syndrome: a pooled study cohort. Occup Environ Med 2013;70:529–37. 10.1136/oemed-2013-101365 23645610 PMC4508843

[21] Salminen S, Kivimaki M, Elovainio M, et al Stress factors predicting injuries of hospital personnel. Am J Ind Med 2003;44:32–6. 10.1002/ajim.10235 12822133

[22] Theorell T, Hasselhorn H On cross-sectional questionnaire studies of relationships between psychosocial conditions at work and health—are they reliable? Int Arch Occup Environ Health 2005;78:517–22. 10.1007/s00420-005-0618-6 15995878

[23] de Jonge J, van Breuklelen GJP, Landeweerd JA, et al Comparing group and individual level assessments of job characteristics in testing the job demand-control model: a multi-level approach. Hum Relat 1999;52:95–122.

[24] North FM, Syme SL, Feeney A, et al Psychosocial work environment and sickness absence among British civil servants: the Whitehall II study.[see comment][erratum appears in *Am J Public Health* 1996;86(8):1093]. Am J Public Health 1996;86:332–40. 10.2105/AJPH.86.3.332 8604757 PMC1380513

[25] Bosma H, Marmot MG, Hemingway H, et al Low job control and risk of coronary heart disease in Whitehall II (prospective cohort) study. BMJ 1997;314:558–65. 10.1136/bmj.314.7080.558 9055714 PMC2126031

[26] Ostry AS, Marion SA, Demers PA, et al Measuring psychosocial job strain with the job content questionnaire using experienced job evaluators. Am J Ind Med 2001;39:397–401. 10.1002/ajim.1030 11323789

[27] Pollack KM, Agnew J, Slade MD, et al Use of employer administrative databases to identify systematic causes of injury in aluminum manufacturing. Am J Ind Med 2007;50:676–86. 10.1002/ajim.20493 17676586

[28] Taiwo OA, Cantley LF, Slade MD, et al Sex differences in injury patterns among workers in heavy manufacturing. Am J Epidemiol 2009;169:161–6. 10.1093/aje/kwn304 18996885 PMC3658145

[29] United States Department of Labor, Employment and Training Administration. Physical demand characteristics. In: Dictionary of occupational titles. 4th edn Appendix C, pp: 1012–1013 Washington, DC: US Department of Labor Employment and Training Administration, 1991.

[30] Bosma H, Peter R, Siegrist J, et al Two alternative job stress models and the risk of coronary heart disease. Am J Public Health 1998;88:68–74. 10.2105/AJPH.88.1.68 9584036 PMC1508386

[31] Karasek R, Brisson C, Kawakami N, et al The Job Content Questionnaire (JCQ): an instrument for internationally comparative assessments of psychosocial job characteristics. J Occup Health Psychol 1998;3:322–55. 10.1037/1076-8998.3.4.322 9805280

[32] Landsbergis P, Theorell T, Schwartz J, et al Measurement of psychosocial workplace exposure variables. Occup Med 2000;15:163–88. 10620790

[33] Stroup W Generalized linear mixed models: modern concepts, methods and applications. Boca Raton, FL: CRC Press, 2012.

[34] Smith PM, Mustard CA Examining the associations between physical work demands and work injury rates between men and women in Ontario, 1990–2000. Occup Environ Med 2004;61:750–6. 10.1136/oem.2003.009860 15317915 PMC1763671

[35] Kubo J, Cullen MR, Cantley L, et al Piecewise exponential models to assess the influence of job-specific experience on the hazard of acute injury for hourly factory workers. BMC Med Res Methodol 2013;13:89 10.1186/1471-2288-13-89 23841648 PMC3727940

[36] Tessier-Sherman B, Cantley LF, Galusha D, et al Occupational injury risk by sex in a manufacturing cohort. Occup Environ Med 2014;71:605–10. 10.1136/oemed-2014-102083 24924313 PMC4145414

[37] Hammar N, Alfredsson L, Johnson JV Job strain, social support at work, and incidence of myocardial infarction. Occup Environ Med 1998;55:548–53. 10.1136/oem.55.8.548 9849542 PMC1757617

[38] Hasselhorn HM, Theorell T, Hammar N, et al Occupational health care team ratings and self reports of demands and decision latitude. Stress Research Reports Stockholm, Sweden: National Institute for Psychosocial Factors and Health, 2004.

[39] Pollack KM, Sorock GS, Slade MD, et al Association between body mass index and acute traumatic workplace injury in hourly manufacturing employees. Am J Epidemiol 2007;166:204–11. 10.1093/aje/kwm058 17485732

[40] Vegso S, Cantley L, Slade M, et al Extended work hours and risk of acute occupational injury: a case-crossover study of workers in manufacturing. Am J Ind Med 2007;50:597–603. 10.1002/ajim.20486 17594716

